# Complete mitochondrial genome of the endangered flower chafer *Osmoderma opicum* (Coleoptera: Scarabaeidae)

**DOI:** 10.1080/23802359.2016.1144104

**Published:** 2016-02-10

**Authors:** Min Jee Kim, Su Yeon Jeong, Jong-Chul Jeong, Sung-Soo Kim, Iksoo Kim

**Affiliations:** a College of Agriculture & Life Sciences, Chonnam National University, Gwangju, Republic of Korea;; b National Park Research Institute, Korea National Park Service, Wonju, Gangwon-do Province, Republic of Korea;; c Research Institute for East Asian Environment and Biology, Seoul, Republic of Korea

**Keywords:** Endangered species, mitochondrial genome*;Osmoderma opicum*

## Abstract

The flower chafer *Osmoderma opicum* (Coleoptera: Scarabaeidae) has been listed in Korea as a class II endangered wild species. The 15 341-bp complete mitochondrial genome (mitogenome) of the species consists of a typical set of genes (13 protein-coding genes [PCGs], two rRNA genes and 22 tRNA genes) and the A + T-rich region, with an arrangement typical of insects. The 761-bp long A + T-rich region of *O. opicum* has a microsatellite-like sequence consisting of (TA)_6_, along with a poly-T (12 bp) and a poly-A (16 bp) stretch, but does not have a longer repeat sequence often found in other Scarabaeidae. Twelve PCGs started with the typical ATN codon, whereas the COI started with AAC, which has been designated as the start codon for the coleopteran suborder Polyphaga. Phylogenetic analysis using 11 PCGs from Scarabaeidae showed that *O. opicum* was placed as the sister to the within-subfamilial species *Myodermum* sp., forming the monophyletic group, the subfamily Cetoniinae, with moderate to high nodal support.

The flower chafer *Osmoderma opicum* (Coleoptera: Scarabaeidae) is distributed throughout Korea and Japan (Won et al. 1998; Bayartogtokh et al. 2012). In Korea, it is found yearly in the north-eastern mountainous areas, from July to August, but with rarity, and thus, is listed as a class II endangered wild animal in Korea (Won et al. 1998). In this study, we sequenced the complete mitochondrial genome (mitogenome) of *O. opicum* to accumulate genetic information of the species. After obtaining the necessary permissions, one adult was collected from a mountain located in Yeongju City, Gyeongsangbuk-do Province, Korea (a detailed description of the administrative district including coordinates is omitted). A voucher specimen was deposited in the National Institute of Biological Resources, Incheon, Korea.

By using the total DNA as template, two long fragments (*COI*–*CytB* and *CytB*–*COI*) were amplified using PCR primers designed in this study (data not shown), and subsequently, 30 short fragments were amplified using the long fragments as templates and the primers designed in this study (data not shown).

The *O. opicum* mitogenome is 15 341 bp in length and includes sets of genes (two rRNAs, 22 tRNAs and 13 protein-coding genes [PCGs]) and a major non-coding 761 bp A + T-rich region (GenBank accession no. KU500641) with arrangements typically found in majority of insects. Currently, a total of 16 mitogenomes are reported from Scarabaeidae, but only three of them are complete with the sequences of A + T-rich region ranging in sizes from 2590 to 5654 bp. The extraordinarily long A + T-rich regions of these species stem either from the presence of seven 82-bp and twenty-eight 117-bp long tandem repeats separated by non-repeat sequences in *Protaetia brevitarsis* (Kim et al. 2014) and ten 88-bp and two 153-bp long tandem repeats abutting each other in *Cheirotonus jansoni* (Shao et al. 2014) or no major repeat units but the presence of a small microsatellite region consisting of TA in *Rhopaea magnicornis* (Cameron et al. 2009). The A + T-rich region of *O. opicum* in the present study is similar to that of *R. magnicornis* in that it only has a microsatellite-like sequence consisting of (TA)_6_, along with a poly-T (12 bp) and a poly-A (16 bp) stretch. Twelve PCGs started with the typical ATN codon (four with ATG, three with ATT and five with ATA), whereas the *COI* gene started with AAC, designated as the start codon for the coleopteran suborder Polyphaga (Sheffield et al. 2008).

We downloaded the available mitogenome sequences of 10 Scarabaeidae from GenBank along with two species of Lucanidae for use as outgroups and 11 (excluding ND1 and ND2 because of unavailability) among 13 PCGs for phylogenetic analysis. Maximum-likelihood (ML) and Bayesian inference (BI) methods were performed using the GTR + I+G model in CIPRES Portal v. 3.1 (Miller et al. 2010). Both the analytical methods presented identical topologies with *O. opicum* as a sister to the within-subfamilial species *Myodermum* sp., but the nodal support for this group was moderate to low (ML, 45%; BI, 0.89) (Figure 1). The subfamily Cetoniinae, in which *O. opicum* is included, formed a strong monophyletic group in the BI analysis (1.0) but was low in the ML method (61%).

**Figure 1.**
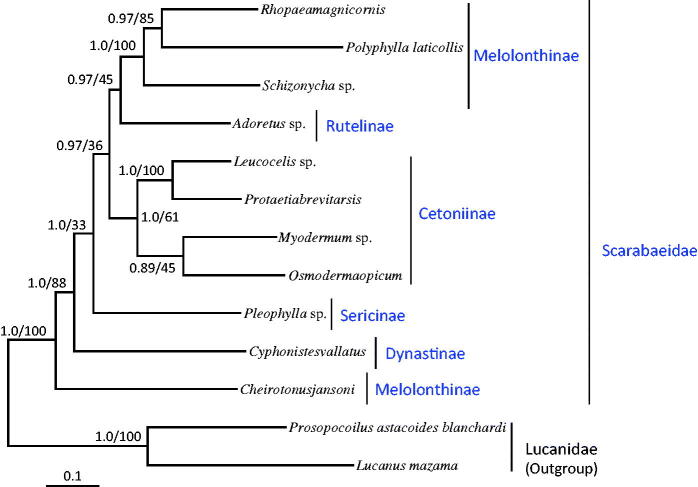
Phylogeny of Scarabaeidae. Bayesian inference (BI) and maximum likelihood (ML) methods produced the same topology based on the concatenated 11 protein-coding genes (PCGs). ND1 and ND2 were excluded in the analysis because of unavailability in several other species of Scarabaeidae. The numbers at each node specify Bayesian posterior probabilities in percent by BI (first value) and bootstrap percentages of 1000 pseudoreplicates by ML (second value). The scale bar indicates the number of substitutions per site. Two species of Lucanidae were utilized as outgroups. GenBank accession numbers are as follows: *Rhopaea magnicornis*, FJ859903; *Polyphylla laticollis*, KF544959; *Schizonycha* sp., JX412739; *Adoretus* sp., JX412788; *Leucocelis* sp., JX412740; *Protaetia brevitarsis*, KJ830749; *Myodermum* sp., JX412847; *Pleophylla* sp., JX412736; *Cyphonistes vallatus*, JX412731; *Cheirotonus jansoni*, KC428100; *Prosopocoilus astacoides blanchardi*, KF364622; and *Lucanus mazama,* NC_013578.
