# Erratum

**DOI:** 10.1080/23802359.2016.1161950

**Published:** 2016-12-23

Kim MJ, Jeong SY, Jeong J-C, Kim S-S, Kim I. (2016). Complete mitochondrial genome of the endangered flower chafer Osmoderma opicum (Coleoptera: Scarabaeidae). *Mitochondrial DNA Part B,* http://dx.doi.org/10.1080/23802359.2016.1144104

When the above article was first published online, Su Yeon Jeong’s affiliation was listed as “National Park Research Institute, Korea National Park Service, Wonju, Gangwon-do Province, Republic of Korea”.

Su Yeon Jeong’s correct affiliation is “College of Agriculture & Life Sciences, Chonnam National University, Gwangju, Republic of Korea”.

This has now been corrected in the online version of this paper.

Taylor & Francis apologises for this error.

